# Clinical outcome, biochemical and therapeutic follow-up in 14 Austrian patients with Long-Chain 3-Hydroxy Acyl CoA Dehydrogenase Deficiency (LCHADD)

**DOI:** 10.1186/s13023-015-0236-7

**Published:** 2015-02-22

**Authors:** Daniela Karall, Michaela Brunner-Krainz, Katharina Kogelnig, Vassiliki Konstantopoulou, Esther M Maier, Dorothea Möslinger, Barbara Plecko, Wolfgang Sperl, Barbara Volkmar, Sabine Scholl-Bürgi

**Affiliations:** Medical University of Innsbruck, Clinic for Pediatrics, Inherited Metabolic Disorders, Anichstrasse 35, 6020 Innsbruck, Austria; University Children’s Hospital, Graz, Austria; University Children’s Hospital, Vienna, Austria; Dr. von Hauner Children’s Hospital, University of Munich, Munich, Germany; University Children’s Hospital, Zürich, Switzerland; University Children’s Hospital, Salzburg, Austria

**Keywords:** Long-chain 3-hydroxy acyl CoA dehydrogenase deficiency, Outcome, Clinical course, Long-term complications, Children

## Abstract

**Background:**

LCHADD is a long-fatty acid oxidation disorder with immediate symptoms and long-term complications. We evaluated data on clinical status, biochemical parameters, therapeutic regimens and outcome of Austrian LCHADD patients.

**Study design:**

Clinical and outcome data including history, diagnosis, short- and long-term manifestations, growth, psychomotor development, hospitalizations, therapy of 14 Austrian patients with LCHADD were evaluated. Biochemically, we evaluated creatine kinase (CK) and acyl carnitine profiles.

**Results:**

All LCHADD patients are homozygous for the common mutation. Three are siblings. Diagnosis was first established biochemically. Nine/14 (64%) were prematures, with IRDS occurring in six. In nine (64%), diagnosis was established through newborn screening, the remaining five (36%) were diagnosed clinically. Four pregnancies were complicated by HELLP syndrome, one by preeclampsia. In two, intrauterine growth retardation and placental insufficiency were reported. Five were diagnosed with hepatopathy at some point, seven with cardiomyopathy and eight with retinopathy, clinically relevant only in one patient. Polyneuropathy is only present in one. Three patients have a PEG, one is regularly fed via NG-tube. Growth is normal in all, as well as psychomotor development, except for two extremely premature girls. In 11 patients, 165 episodes with elevated creatine kinase concentrations were observed with 6-31 (median 14) per patient; three have shown no elevated CK concentrations. Median total carnitine on therapy was 19 μmol/l (range 11-61). For 14 patients, there have been 181 hospitalizations (median 9 per patient), comprising 1337 in-patient-days. All centres adhere to treatment with a fat-defined diet; patients have between 15% and 40% of their energy intake from fat (median 29%), out of which between 20% and 80% are medium-chain triglycerides (MCT) (median 62%). Four patients have been treated with heptanoate (C7).

**Conclusion:**

Our data show LCHADD outcome can be favourable. Growth and psychomotor development is normal, except in two prematures. Frequency of CK measurements decreases with age, correlating with a decreasing number of hospitalizations. About 50% develop complications affecting different organ systems. There is no relevant difference between the patients treated in the respective centers. Concluding from single case reports, anaplerotic therapy with heptanoate should be further evaluated.

## Background

Long-chain 3-hydroxy acyl CoA dehydrogenase deficiency (LCHADD) (OMIM #609016) is an autosomal recessively inherited disorder of long-chain fatty acid oxidation with an estimated overall frequency of 1:50,000, first described in 1989 in children presenting with hypoketotic hypoglycemia and lethargy after periods of fasting, often associated with febrile infections and gastroenteritis [1,2]. LCHAD is part of the mitochondrial trifunctional protein (MTP) and specific for the metabolism of C12-C16 chain-length fatty acid compounds. LCHADD leads to an accumulation of toxic β-oxidation intermediates causing immediate symptoms as well as long-term complications. It was included in the Austrian Newborn Screening Program in April 2002. An association between maternal HELLP syndrome, prematurity and fetal LCHADD has been previously published [3-5]. Clinical symptoms mainly develop during episodes of illness or fasting and affect organs needing long-chain fat as primary energy source such as heart and skeletal muscle [6-8]. Impaired glucose production during catabolism results in hypoketotic hypoglycemia [9]. Therapy aims at defining the intake of exogenous long-chain fatty acids as well as preventing catabolic episodes, which lead to energy mobilization out of endogenous fat [10]. Anaplerotic therapy with e.g. heptanoate (C7) is based on the concept of providing an odd-carbon numbered substrate for the citric acid cycle and the electron transport chain that bypasses the deficient fatty acid oxidation (FAO) enzymes in order to enhance ATP production. Significant relief of FAOD-related clinical symptoms such as hypertrophic cardiomyopathy, congestive heart failure, hepatomegaly and muscle weakness has been reported in VLCADD patients with a diet containing 30-35% of total caloric intake as heptanoate [11].

Although LCHADD is a fatty acid oxidation disorder with significant morbidity, information on the development and outcome is still rather limited.

Therefore, we collected data of the 14 living Austrian patients with LCHADD, including clinical status, biochemical parameters, therapeutic regimens and outcome.

## Methods

### Study population

We retrospectively collected data of 14 living patients with LCHADD, cared for in four Austrian (Graz, Innsbruck, Salzburg, Vienna) and one German (Munich) metabolic center. Data of all 14 patients were reviewed at their respective metabolic center from birth until October 2013. Thus, follow-up times were between 0.9 and 15.4 years (median 7.8 years, mean 6.9 years). The 14 patients come from 11 families and include 3 siblings of consanguineous parents (Patient 5, 13, 14). Details of the study cohort are displayed in Table 1. All patients have biochemically confirmed LCHADD and are homozygous for the common mutation c.1528G>C. In ten, the diagnosis was confirmed enzymatically in fibroblasts.

**Table 1:** **Background data of the study cohort**

**Patient (metabolic center)**	**Gender**	**Current age (y)**	**Age at diagnosis**	**NBS+**	**Decompensation at diagnosis.**	**Pregnancy**	**Birth mode**	**GA**
**Patients born before introduction of LCHADD into NBS program, diagnosed clinically**								
**1 (Munich)**	M	15.3	5 m	no*	yes	HELLP	CS	37 weeks
**2 (Innsbruck)**	M	14.1	23 m	no*	yes	normal	CS	38 weeks
**3 (Graz)**	F	12.2	3 m	no*	yes	normal	vaginal	Term
**Patients born after introduction of LCHADD into NBS program (group 1-3):**								
**Group 1: patients showing symptoms before NBS results were available:**								
**4 (Innsbruck)**	F	7.8	15 d	yes	(yes)	IUD	CS	32 weeks
**5 (Vienna)**	M	7.3	15 d	yes	(yes)	normal	vaginal	35 weeks
**6 (Innsbruck)**	M	2.7	15 d	yes	(yes)	IUD	CS	32 weeks^#^
**Group 2: patients with false negative NBS results:**								
**7 (Graz)**	M	10.6	4 m	no**	yes	HELLP	CS	31 weeks^#^
**8 (Salzburg)**	M	5.2	5 m	no**	yes	HELPP	CS	29 weeks^#^
**Group 3: patients with positive NBS results, asymptomatic:**								
**9 (Vienna)**	F	10.8	1 d	Yes	no	normal	vaginal	Term
**10 (Vienna)**	F	9.5	6 d	Yes	no	twin pregnancy	CS	29 weeks^#^
**11 (Graz)**	F	3.7	1 d	yes	no	HELLP	CS	32 weeks^#^
**12 (Vienna)**	M	2.8	10 d	Yes	no	preeclampsia	CS	34 weeks
**13 (Vienna)**	F	2.7	1 d	Yes	no	path. CTG	CS	25 weeks^#^
**14 (Vienna)**	M	0.9	2 d	Yes	no	normal	vaginal	40 weeks
**Summary for the 14 LCHADD patients:**								
	8 M/6 F	median: 7.6 y	median:15 d	NBS + 9/14	8/14	4/14 HELLP	CS 10/14	9/14 preterm 64%; 6/9 IRDS
		range: 0.9-15.3	range: 1d-20 m	64%	57%	1/14 preecl.	71%	

Table shows patient number and treating center, gender, current age (referring to October 2013), age at diagnosis, newborn screening result, decompensation at time of diagnosis, pregnancy, birth mode, and gestational age.

Patients are grouped by mode of diagnosis, either born before or after introduction of LCHADD into NBS program.

(NBS+) diagnosis through newborn screening; (GA) gestational age; (C7) therapy with triheptanoate; (M) male; (F) female; (y) years; (m) months; (d) days; (IUD) intrauterine dystrophy, following placental insufficiency; (HELLP) hemolysis, elevated liver enzymes, low platelet count syndrome; (path. CTG) pathological CTG, bradycardia; (CS) Caesarean section. *born before LCHADD was included into Austrian Newborn Screening Program, **newborn screening false negative, probably due to prematurity; ^#^IRDS present; (IRDS) infant respiratory distress syndrome.

### Data collection

We collected clinical information by review of medical records. Besides epidemiological data (Table 1), we evaluated: growth, short and long-term complications, number of hospital admissions and length of stay (in days per year); creatine kinase concentrations (peak levels) as a marker for rhabdomyolysis, and two acyl carnitine profiles per patient (one at diagnosis and last one available). Fat intake and quality of fats was evaluated with available dietary protocols; and information on PEG/NG tube feeding, late evening feeds, bolus before sports, as well as concomitant medications was retrieved.

The study was conducted with the approval of the ethics committee of the Medical University of Innsbruck. Research was carried out in compliance with the Helsinki Declaration. Parents gave their consent for this retrospective data collection.

### Statistics

As the cohort is significant for a rare disorder but rather small in principle, only descriptive statistical analyses were performed.

## Results

### Diagnosis and onset of symptoms

Nine/14 patients were diagnosed by newborn screening, three/nine showing clinical symptoms before results were available (Patient 4, 5, and 6) (Table 1). In two additional ones (Patient 7 and 8) newborn screening was false negative, probably due to prematurity and L-carnitine supplementation (Table 1). The other three (Patient 1, 2, 3) were born before the inclusion of LCHADD into the newborn screening panel and were detected through metabolic decompensation at 5 months, 23 months, and 3 months of age, respectively (Table 1) with metabolic acidosis, hepatopathy, cardiomyopathy, rhabdomyolysis; and coma in Patient 2.

Nine/14 patients were born prematurely (Table 1), postpartally, six of them showed IRDS, and three inguinal hernia (Patient 6, 7, 11). Four pregnancies were complicated by HELLP syndrome and one by preeclampsia (Table 1).

### Clinical signs – short- and long-term organ involvement

All patients are in a stable clinical condition and have normal growth. At some point, 5/14 patients have shown hepatopathy defined as a sonographic finding and elevated liver enzyme tests (range 0-23 months, median 4), and 7/14 cardiomyopathy defined as fraction shortening (FS) <25% and/or ejection fraction (EF) <50% in at least one echocardiographic screening (range 3-156 months, median 5). Retinopathy has been reported in 8/14, however, only one (Patient 3) suffers from severe impairment of vision. Polyneuropathy has been reported only in the oldest patient so far (Patient 1), with onset at 9 years (Table 2).

**Table 2:** **Clinical signs – short and long-term organ involvement in the 14 Austrian patients with LCHADD and when firstly diagnosed in months of age**

**Patient**	**Current age (y)**	**Hepatopathy**	**CMP**	**Retinopathy**	**NG/PEG**	**Late night feeds**
**Patients born before introduction of LCHADD into NBS program, diagnosed clinically**						
**1**	15.3		156 m	108 m	PEG 24 m	X*
**2**	14.1	23 m	23 m	23 m		X
**3**	12.2	3 m	3 m	24 m		
**Patients born after introduction of LCHADD into NBS program (group 1-3):**						
**Group 1: patients showing symptoms before NBS results were available:**						
**4**	7.8					
**5**	7.3			39 m		
**6**	2.7		9 m			
**Group 2: patients with false negative NBS results:**						
**7**	10.6	4 m	4 m	42 m		
**8**	5.2	5 m	5 m	38 m	PEG 12 m	X*
**Group 3: patients with positive NBS results, asymptomatic:**						
**9**	10.8			50 m		
**10**	9.5		4 m	56 m	PEG 11 m	X
**11**	3.7				NG 7 m	
**12**	2.8					X
**13**	2.7	neonatally				X
**14**	0.9					X
**Summary for the 14 LCHADD patients:**						
		36%	50%	57%	29%	50%

Patients grouped by mode of diagnosis (see Table 1). Cardiomyopathy in Patient 1, 2, 3, 6, 7, 8, 10 is defined as fractional shortening (FS) <25 and/or ejection fraction (EF) < 50 in at least one echocardiographic screening, Patient 3 showed a prolonged QTc interval of 0.45 msec (0.42 +/- 0.02); retinopathy as described in fundoscopy reports, hepatopathy defined as a sonographic finding and elevated liver enzyme tests. Current age (referring to October 2013), (y) years; (m) months; (CMP) cardiomyopathy; (NG) nasogastric tube; (PEG) percutaneous endoscopic gastrostomy. Late evening meal, usually 22:00; X* - continuous night drip feeding via PEG.

### Creatine kinase

In 11 patients, there have been 165 episodes with elevated creatine kinase concentrations (sign of muscle involvement/rhabdomyolysis, defined as CK levels >1,000 U/l, range 1,000-95,000, median 40,381). The range per patient is 6-31 episodes (median 14) (Table 3). In three patients no elevated CK concentrations (>1,000 U/l) have been measured so far. For 14 patients, there are a total of 722 CK (range 9-132 per patient) measurements available (Table 3).

**Table 3:** **Features of creatine kinase in 14 patients with LCHADD - patients grouped by mode of diagnosis (see Table** 1 **and** 2 **)**

**Patient**	**n CK**	**max CK (in U/l)**	**max CK (age)**	**n CK >1,000 (in U/l)**
**1**	57	40,381	16.2 y	19
**2**	127	34,160	4.2 y	31
**3**	36	39,501	5.8 y	10
**4**	132	10,170	1.9 y	19
**5**	67	8,876	3.9 y	17
**6**	15	299	0.8 y	0
**7**	52	62,216	2.7 y	6
**8**	69	73,260	0.9 y	27
**9**	22	95,000	2.7 y	8
**10**	71	67,500	2.6 y	14
**11**	29	68,600	0.6 y	6
**12**	26	12,000	2.1 y	8
**13**	9	449	1.7 y	0
**14**	10	725	2 d	0
**Median**	44	40,381	2.35 y	14

(n CK) number of CK measurements, (max CK (in U/l)) highest measured CK, (max CK (age)) age when highest CK was measured, (n CK > 1,000) number of CK measurements >1,000 U/l, (y) years.

### Total carnitine and acyl carnitine profiles

Carnitine state and acyl carnitine profiles were collected at the time of diagnosis and last analysis available (Table 4). At the time of diagnosis, which ranges from 1 day to 20 months (median 15 days), median total carnitine was 44 μmol/l (range 10-118), free carnitine 19 μmol/l (range 3-97), C16OH 0.4 μmol/l (range 0.2-1.1), C18:1OH 0.5 μmol/l (range 0.1-1.2), and C18OH 0.4 μmol/l (range 0.2-0.8). Three prematures were being treated with L-carnitine. At the time of last acyl carnitine profile available, which ranges from 0.3 to 15 years (median 7), median total carnitine was 19 μmol/l (range 11-61), free carnitine 10 μmol/l (range 6-46), C16OH 0.3 μmol/l (range 0.1-0.5), C18:1OH 0.4 μmol/l (range 0.1-0.7), and C18OH 0.3 μmol/l (range 0.1-1.0). No patient is being treated with L-carnitine. In summary, concentrations are generally lower after diagnosis is known. There is no correlation between acyl carnitine concentrations and age.

**Table 4:** **Acyl carnitine profiles from 14 Austrian LCHADD patients at diagnosis and at last follow-up**

	**Acylcarnitine profile at diagnosis**						**Acylcarnitine profile at last follow-up**					
**Patient**	**Age at diagn.**	**TC**	**C0**	**C16OH**	**C18:1OH**	**C18OH**	**Age at last follow-up**	**TC**	**C0**	**C16OH**	**C18:1OH**	**C18OH**
1	5 m	-	5	0.57	1.19	0.39	15.0 y	-	39	0.19	0.22	-
2	23 m	21	3	0.17	0.08	-	13.5 y	18	11	0.16	0.17	0.23
3	3 m	-	11	0.69	0.54	-	9.0 y	32	25	0.01	0.05	0.10
4	*15 d	45	27	0.30	0.50	0.20	7.2 y	14	9	0.37	0.44	1.00
5	15 d	42	20	0.20	0.16	0.40	6.8 y	18	10	0.11	0.49	0.54
6	*15 d	91	45	0.48	0.62	0.50	2.1 y	15	7	0.42	0.66	0.78
7	4 m	10	5	0.24	0.66	-	9.0 y	45	37	-	0.01	0.01
8	5 m	17	9	0.98	0.74	-	3.6 y	48	28	0.25	0.11	0.24
9	1 d	13	8	0.82	-	0.49	8.6 y	11	6	0.31	0.65	0.41
10	6 d	118	97	0.35	-	0.80	8.7 y	17	10	0.12	0.10	0.20
11	1 d	77	33	-	-	-	3.2 y	61	46	0.13	-	-
12	*10 d	103	56	0.25	0.37	0.34	2.2 y	-	-	0.31	0.42	0.35
13	1 d	39	18	0.21	0.27	0.30	2.1 y	20	10	0.52	0.69	0.95
14	2 d	68	32	1.1	0.41	0.69	0.3 y	22	10	0.53	0.40	0.47
Median	15 d	44	19	0.35	0.50	0.40	7.0	19	10	0.25	0.40	0.30
Range	1d-20 m	10-118	3-97	0.17-1.1	0.08-1.19	0.2-0.8	0.3-15.0	11-61	6-46	0.01-0.53	0.01-0.69	0.01-1.0

Patients grouped by mode of diagnosis (see Table 1 and 2).

Concentrations in μmol/l. (TC) total carnitine; (C0) free carnitine, (C16OH) 3-hydroxyhexadecanoylcarnitine; (C18:1OH) 3-hydroxyoctadecenoylcarnitine, (C18OH3)-hydroxyoctadecanoylcarnitine; (d) days; (m) months; (y) years.

Reference values are: TC 7-70 μmol/l, C0 6-54 μmol/l, C16OH < 0.12 μmol/l, C18:1OH < 0.16 μmol/l, C18OH < 0.13 μmol/l, (-) no values given in laboratory report.

*patients receiving carnitine therapy as prematurity treatment.

### PEG/NG tube feeding/late evening feeds/bolus before sports/concomitant medication

Ten/14 patients eat self-sufficiently, three have a PEG (Patient 1, 8, and 10) and one is being regularly fed via nasogastric tube (Patient 11), all four presented with regular vomiting and refusal of oral feeds with somatic reasons being excluded. Patient 10 has a reflux diagnosed with 10 months. PEG were installed at 12 (Patient 8), 11 months (Patient 10) and 24 months (Patient 1) of age and have been in place for 4.7, 8.6 and 13 years, respectively. Patient 11 still shows very little interest in food and receives most of her caloric intake in form of a MCT formula (Monogen®) via NG tube. Patient 8 has improved from absolute refusal of oral feeds to occasional savoring of food samples by seeing a speech therapist. Of the 14 patients, six are reported to have late evening meals regularly, usually around 22:00, in additional two (Patient 1, 8), PEG is used for continuous night drip feeding (Table 2).

Only Patient 2 is reported to regularly take a calorie bolus before exercise (sports) [12], and only two receive additional long term medication; Patient 7 due to allergic asthma, and Patient 1 cardiomyopathy treatment (diuretics and ramipril).

### Hospitalizations

There have been 181 hospitalizations for 14 patients (range 2-34, median 9), comprising 1337 in-patient-days (range per patient 23-247, median 78) (Table 5). Except for three patients, the first hospitalization is the longest, often involving LCHADD unrelated problems, like e.g. prematurity. With increasing age, hospitalizations become less frequent (Table 6).

**Table 5:** **Number of hospitalizations in 14 Austrian LCHADD patients**

**Patient**	**1st hospitalization and time of LCHADD diagnosis (age)**	**Length of 1st hospitalization (days)**	**Number of hospitalizations including 1st hospitalization**	**Length of hospitalizations (total days)**	**Range (days)**	**Median (days)**
**1**	5 months	16	8	79	1-23	7
**2**	23 months	26	18	76	1-26	2
**3**	3 months	22	7	48	2-22	3.5
**4**	GA 32 weeks	29	34	114	1-29	3
**5**	GA 35 weeks positive family history		17	77	1-10	3
**6**	GA 32 weeks	36	6	16	2-36	3
**7**	GA 31 weeks; Dg. LCHADD 4 months	46	17	168	2-46	4
**8**	GA 29 weeks	26	22	181	2-29	4
**9**	NBS		6	22	1-9	4
**10**	GA 29 weeks	89	23	247	1-89	8
**11**	GA 32 weeks	55	10	94	1-55	3
**12**	GA 34 weeks	23	6	27	3-23	4
**13**	GA 25 weeks	91	2	94	3-91	47
**14**	NBS positive family history	6	2	23	6-17	11.5
**Median**		27.5	9	78		
**Range**		6 – 91	2 – 34	16 – 247		

Patients grouped by mode of diagnosis (see Table 1 and 2).

As the first hospitalization is often combined with other LCHADD independent causes, e.g. prematurity, it is listed separately. Except for Patient 7, first hospitalization and time of diagnosis are the same.

**Table 6:** **Hospitalizations; days/stays per patient (y-axis) and year of life (x axis; Y1, year 1 defined as 0-12 months; Y2, year 2 defined as 12-24 months, etc.)**

**Patient**	**Y1**	**Y2**	**Y3**	**Y4**	**Y5**	**Y6**	**Y7**	**Y8**	**Y9**	**Y10**	**Y11**	**Y12**	**Y13**	**Y14**	**Y15**
**1**	39/2				1/1		3/1					19/1		11/2	6/1
**2**	0/0	26/1	13/4	14/3	20/7	3/2	0	0	0	0	1/1	0	0	0	
**3**	33/3	5/2	4/1			3/1	3/1								
**4**	45/7	26/6	18/3	21/7	4/2	10/3	13/4	6/2							
**5**	11/3	18/5	2/1	13/2	5/1	6/2	22/3								
**6**	50/5	2/1													
**7**	109/5	22/4	2/1		12/3	3/1	20/3								
**8**	94/8	59/8	20/5	5/2	3/1										
**9**	4/1	5/1	9/1	0	0	5/2	0	0	0	4/1					
**10**	176/13	48/4	14/3	4/1	0	0	0	5/2	0						
**11**	84/6	1/1	3/1	6/2											
**12**	0	10/2	17/4												
**13**	91/1	0	3/1												
**14**	23/2														

### Long-term management - diet

At the time of last dietary protocol available, which ranges from 0.9 to 15 years (median 5.6), all 14 patients are on a fat defined diet, with a median fat intake from total energy of 29% (range 15 – 40), and thereof a median MCT intake of 62% (range 20 – 80). For supplementation of long-chain essential fatty acids, 13 receive walnut oil (median 0.3 g/kg/d, range 0.1 – 0.8), Patient 1 does not. Additional DHA supplementation is given to 10/14 patients.

### Heptanoate (C7)

Four patients have been treated with heptanoate (Patient 2,4,6,8), which they have been receiving for different periods of time (9.4 years, 7 years, 13 months and 13 months). In Patient 2, the dosage equals 0.6 g/kg/day, in Patient 4, 0.8 g/kg/day, in Patient 6, 0.75 g/kg/day, in Patient 8, 0.6 g/kg/day (Table 7). Patient 8 discontinued C7 after 13 months, because no essential difference was appreciated by parents and care takers.

**Table 7:** **Dietary treatment in the 14 Austrian patients with LCHADD, calculated from a dietary protocol**

**Patient**	**Age at evaluation**	**Body weight (kg)**	**Total fat intake**	**% MCT of fat in diet**	**Walnut oil (in ml and g/kg/d)**	**Heptanoate (C7) (in ml and g/kg/d)**	**C7 since**	**DHA suppl.**	**kcal/d**
**1**	15.0 y	72,6	30%	20%	0 ml	-		+267 mg	3000
**2**	13.5 y	55.5	40%	50%	5 ml = 0.10	30 ml = 0.60	9.4 y	-	1800
**3**	10.0 y	29.7	33.5%	72%	10 ml = 0.34	-		+89 mg	1700
**4**	7.2 y	24.9	25%	52%	10 ml = 0.40	20 ml = 0.80	7 y	-	1738
**5**	7.3 y	29.0	27%	56%	3 ml = 0.10	-		*200 mg	1653
**6**	2.7 y	13.3	36%	47%	6 ml = 0.45	10 ml = 0.75	13 m	-	953
**7**	10.6 y	36.9	29%	69%	10 ml = 0.27	-		+89 mg	1900
**8 #**	4.0 y	17.4	15%	80%	10 ml = 0.57	10 ml = 0.60	13 m	-	1452
**9**	10.7 y	25.0	21%	68%	2 ml = 0.08	-		*200 mg	1850
**10**	9.3 y	25.1	28%	70%	13 ml = 0.52	-		*100 mg	1782
**11**	3.7 y	15.4	25%	64%	13 ml = 0.84	-		+89 mg	1300
**12**	2.8 y	15.3	30%	63%	4 ml = 0.26	-		*100 mg	1377
**13**	2.7 y	11.1	30%	61%	3 ml = 0.27	-		*150 mg	1166
**14**	0.9 y	8.62	33%	65%	3 ml = 0.35	-		*150 mg	862
Median	5.6 y		28.5%	62%	0.34				
Range	0.9 – 15.0		15 – 40	20 – 80	0.08 – 0.84				

Amount of fat in diet, % MCT (middle chain triglycerides) in diet, walnut oil and C7 intake in ml and g/kg/day, *DHA supplementation: Key Omega® 4 g sachet, containing 100 mg DHA; + 1 fish oil capsule, containing 89 mg DHA. #Patient 8 received C7 from age 3.75 until age 5 in an amount of 10 ml/d (=0.6 g/kg/d). (y) years; (m) months.

**Figure 1:**
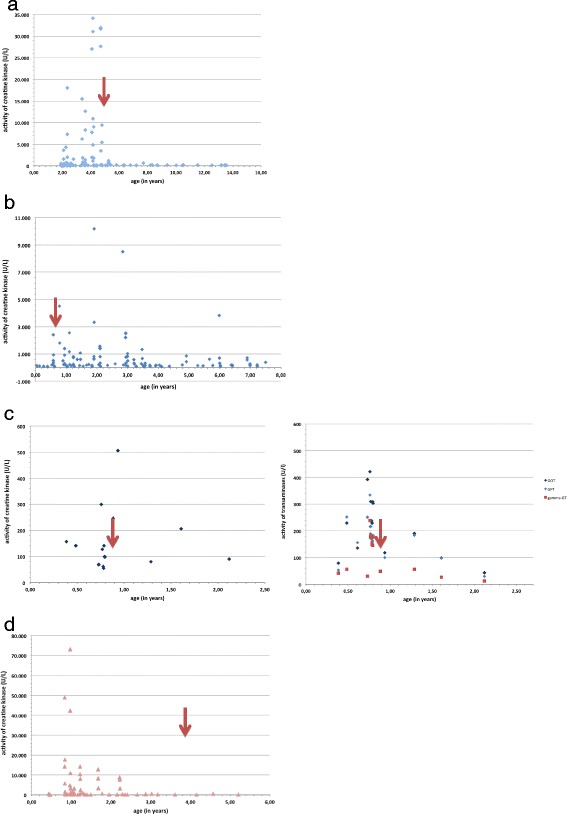
**CK and liver enzymes in the four patients treated with heptanoate (start marked with red arrows). a**: CK concentrations in Patient 2 before and after the introduction to therapy with heptanoate. **b**: CK concentrations and start of heptanoate in Patient 4. **c**: CK concentrations (left) and liver enzymes tests (right) before and after introduction of heptanoate in Patient 6. **d**: CK concentrations and start of heptanoate in Patient 8.

After heptanoate was started, we observed a better clinical stability and no more CK peaks in Patient 2 [12], therefore we compared outcome of the patients that receive heptanoate (Figure 1). No statistical differences can be seen, however Patients 2,4,6 appear clinically more stable, and parents will not discontinue heptanoate treatment. Patient 4 did show episodes of rhabdomyolysis (CK max 10,170 U/l), but her CK levels were never as high (Figure 1). Before heptanoate was started, Patient 6 needed repeated hospitalizations during infections with marked hepatopathy, he now seems clinically more stable. CK concentrations have not shown significant changes – there never was a CK measured above 1,000 U/l, but the transaminases seem to stabilize (see Figure 1). In Patient 8 with failure to thrive, heptanoate was started with 45 months, and discontinued after 13 months because of stomache aches and diarrhea, which he also had prior to C7 supplementation.

## Discussion

In this retrospective data analysis of the 14 living Austrian patients with LCHADD, we wanted to collect and evaluate clinical status, biochemical parameters, therapeutic regimens and outcome.

In literature, we found a total of 196 cases of LCHADD reported [7,8,13-27]; it must be assumed that some patients are reported repeatedly (e.g. Gillingham [10,16-18]). DenBoer et al. looked at similar parameters as we did (e.g. clinical, biochemical and therapeutic follow-up of patients with isolated LCHADD) [7]. Other studies either included other fatty acid oxidation disorders or focused on more narrow and specific questions concerning patient development, such as for example growth, ophtalmological findings or metabolic control during exercise [18,20,27].

In contrast to data evaluation out of patient charts, DenBoer et al. sent out a standardized questionnaire to referring physicians of 61 unselected patients with LCHADD, 50 questionnaires (82%) were returned [7]. Our 14 patients’ cohort reflects all living Austrian LCHADD patients, 8 male (57%) and 6 female (43%) patients, DenBoer’s cohort 23 male (46%) and 27 female (54%) patients.

In our cohort, the median ***age of presentation*** was 15 days (range: 1 day to 23 months) for the whole cohort and 5 months (range 3 to 23 months) for the 5 patients not detected by newborn screening versus mean 5.8 months (range: 1 day to 26 months) in DenBoer’s cohort [7]. Four/14 ***pregnancies*** (28%) were complicated by HELLP syndrome in comparison to 15% (7/47 pregnancies) observed by DenBoer [7].

All of DenBoer’s patients were diagnosed clinically, as LCHADD had not been included the Dutch ***Newborn Screening Program*** at the time the study was conducted. Patients showed hypoketotic hypoglycemia in 78%, chronic liver disease, failure to thrive, feeding difficulties/hypotonia in 22%. In Austria, LCHADD was included in the NBS program in 2002, only patients that were born before (n = 3) or showed false negative results due to prematurity (n = 2) were diagnosed clinically. However, from the ones with positive NBS results in our cohort, 3/9 patients were symptomatic before NBS results were available. However, they were not as severely diseased as the patients reported by DenBoer: Of these 39/50 presenting with acute metabolic derangement, 22 (56%) were comatous, 15 (38%) had seizures, 9 (23%) apneic spells, 8 (21%) cardiorespiratory arrest, 7 (18%) arrhythmias and 3 (9%) died. These symptoms were not seen in our follow-up. In our cohort, only Patient 2 was found to be comatous at the point of first metabolic derangement.

***Long-term complications*** are present in 36%-57% (Table 2). In summary, the 5 children diagnosed clinically (the three born before NBS was introduced) (Patient 1,2,3) and the two with false negative NBS results (Patient 7,8), showed cardiomyopathy mostly at time of diagnosis. Four of these five diagnosed clinically have had hepatopathy, but only one/nine patients diagnosed by NBS. Retinopathy is present in the older patients, regardless of mode of diagnosis. However, as expected in this age group, the impact of retinopathy and polyneuropathy is low, with only one patient showing vision impairment and one patient having polyneuropathy.

We did not focus on deceased LCHADD patients and do not have data concerning LCHADD mortality in Austria. The two deceased LCHADD patients close to the cohort were the the older sister of Patient 1 and the older brother of Patients 5, 13 and 14.

***Creatine kinase (CK)*** is an enzyme expressed in ATP consuming tissues, e.g. skeletal muscle and retinal photoreceptor cells, and is used as a marker of myocardial infarction, rhabdomyolysis, muscular dystrophy, autoimmune myositis and in acute renal failure [28,29]. In LCHADD patients it is used to determine rhabdomyolysis during metabolic derangement. CK determinations in our cohort (Table 3) show that CK is a reliable but unspecific marker. Episodes of rhabdomyolysis decrease with age, pointing to less infectious illnesses as expected in every child growing older.

***Acyl carnitine profile analysis*** is essential in the diagnosis of LCHADD. Acyl carnitine abnormalities in our cohort were consistent with those previously reported for LCHADD. Total long-chain acyl carnitines are used for clinical purposes [17], and should not exceed 2 μmol/l. Our profiles have values compatible with this recommendation. L-carnitine substitution in patients with LCHADD is no longer recommended even when carnitine is low, due to the proposed increased generation of long-chain acyl carnitines and arrhythmogenic effect from accumulating hydroxylated long-chain acyl CoA esters [30]. None of our patients receives carnitine.

So far, ***treatment*** recommendations for disorders of long-chain FAOD are based on expert opinion [31]. In 2009, a consensus was published [8], recommending the total dietary fat content in LCHADD patients to be 25-30% of total energy intake, with 20-25% as MCT and 5-10% as LCT. Most of our patients are within the range recommended, however, in some MCT intake clearly deviates with a median of 62%. Even though not proven in humans so far, on a cautious note, this could lead to endogenous fatty acid elongation and hepatopathy [30]. More recent data suggest that the fat modification in the diet is less important than the avoidance of catabolic state (e.g., infections, physical exertion) [30]. Therefore, fat restriction is becoming less strict in clinical practice. It is recommended to give a high energy bolus before exercise [12,18]. However, in our cohort only Patient 2 is reported to regularly do that, taking a carbohydrate bolus [12].

Spiekerkötter et al. reported continual overnight nasogastric tube feeding in 14% of their LCHADD patients [8], which is similar to our cohort with 2/14 (14.1%). There are no recommendations or indications for NG/PEG in LCHADD. The decision remains a clinical one, when patients refuse feedings or the fasting intervals are prolonged. In our opinion, it should be an achievable goal to manage children with LCHADD without the need of a NG/PEG.

We tried to use ***hospitalization*** number and length in LCHADD patients as an indirect marker for metabolic stability. It needs to be said, that LCHADD parents are aware of the need of early intervention in case of catabolic episodes. Therefore, short hospitalizations are more frequent in this patient group. In summary, for our cohort, except for 3 patients, the first hospitalization is the longest (ranging 6 – 91 days, median 27.5), often involving LCHADD unrelated problems, like e.g. prematurity. With increasing age, hospitalizations become less frequent, indicating less infections with increasing age.

As anaplerotic substance, ***heptanoate (C7)*** supplementation has also been a therapeutic approach in LCHADD. So far, studies in animal and cell models [32,33] and small clinical trials [11,34,35] have given beneficial proof of impact on hepatic, cardiac and muscular symptoms. The longest published observational period is of 61 months [35], there are no controlled studies on the long-term effects so far. We can contribute information on C7 supplementation over a period of almost 10 years. However, we were not able to find a good marker to evaluate the impact of heptanoate in the clinical course. Our data (Figure 1 and Tables 5 and 6) show that patients stabilize with age, which we attributed to the anaplerotic effect of heptanoate, but maybe just came with increasing age (less infections). However, there is no obvious difference between patients with and without C7 supplementation. To clarify the effect of heptanoate, a placebo-controlled trial should be conducted.

## Conclusion

Our data show that the outcome of LCHADD can be favourable. Growth and psychomotor development is normal in all patients, except in two prematures. Frequency of CK measurements decreases with age, which correlates with a decreasing number of hospital stays and out-patient visits as well. About 50% of patients develop complications affecting different organ systems. However, so far the clinical relevance is low in this pediatric age group. There is no relevant difference between the patients treated in the respective centers. Concluding from single case reports, anaplerotic therapy with heptanoate should be further evaluated, e.g. with conduction of a placebo-controlled trial.

## Abbreviations

C7: Heptanoate
CK: Creatine kinase
CoA: Coenzyme A
EF: Ejection fraction
FS: Fraction of shortening
FAOD: Fatty acid oxidation disorders
GA: Gestational age
HELLP: Hemolysis, elevated liver enzymes, low platelet count
IRDS: Infant respiratory distress syndrome
LCHADD: Long-chain 3-hydroxy acyl CoA dehydrogenase deficiency
LCT: Long-chain triglycerides
MCT: Medium-chain triglycerides
NBS: Newborn screening
NG-tube: Nasogastric tube
PEG: Percutaneous endoscopic gastrostomy
VLCADD: Very long-chain acyl CoA dehydrogenase deficiency

## References

[1] Wanders RJ, Duran M, IJlst L, de Jager JP, van Gennip AH, Jakobs C (1989). Sudden infant death and long-chain 3-hydroxyacyl-CoA dehydrogenase. Lancet.

[2] Wanders RJ, IJlst L, van Gennip AH, Jakobs C, de Jager JP, Dorland L (1990). Long-chain 3-hydroxyacyl-CoA dehydrogenase deficiency: identification of a new inborn error of mitochondrial fatty acid beta-oxidation. J Inherit Metab Dis.

[3] Treem WR, Rinaldo P, Hale DE, Stanley CA, Millington DS, Hyams JS (1994). Acute fatty liver of pregnancy and long-chain 3-hydroxyacyl-coenzyme A dehydrogenase deficiency. Hepatology.

[4] Strauss AW, Bennett MJ, Rinaldo P, Sims HF, O’Brien LK, Zhao Y (1999). Inherited long-chain 3-hydroxyacyl-CoA dehydrogenase deficiency and a fetal-maternal interaction cause maternal liver disease and other pregnancy complications. Semin Perinatol.

[5] Shekhawat P, Bennett MJ, Sadovsky Y, Nelson DM, Rakheja D, Strauss AW (2003). Human placenta metabolizes fatty acids: implications for fetal fatty acid oxidation disorders and maternal liver diseases. Am J Physiol Endocrinol Metab.

[6] Ørngreen MC, Nørgaard MG, Sacchetti M, van Engelen BGM, Vissing J (2004). Fuel utilization in patients with very long-chain acyl-coa dehydrogenase deficiency. Ann Neurol.

[7] den Boer MEJ, Wanders RJA, Morris AAM, IJlst L, Heymans HSA, Wijburg FA (2002). Long-Chain 3-Hydroxyacyl-CoA dehydrogenase deficiency: clinical presentation and follow-up of 50 patients. Pediatr.

[8] Spiekerkoetter U, Lindner M, Santer R, Grotzke M, Baumgartner MR, Boehles H (2009). Management and outcome in 75 individuals with long-chain fatty acid oxidation defects: results from a workshop. J Inherit Metab Dis.

[9] Spiekerkoetter U, Ruiter J, Tokunaga C, Wendel U, Mayatepek E, Wijburg FA (2006). Evidence for impaired gluconeogenesis in very long-chain acyl-CoA dehydrogenase-deficient mice. Horm Metab Res.

[10] Gillingham M, Van Calcar S, Ney D, Wolff J, Harding C (1999). Dietary management of long-chain 3-hydroxyacyl-CoA dehydrogenase deficiency (LCHADD). A case report and survey. J Inherit Metab Dis.

[11] Roe CR, Sweetman L, Roe DS, David F, Brunengraber H (2002). Treatment of cardiomyopathy and rhabdomyolysis in long-chain fat oxidation disorders using an anaplerotic odd-chain triglyceride. J Clin Invest.

[12] Karall D, Mair G, Albrecht U, Niedermayr K, Karall T, Schobersberger W, et al. Sports in LCHAD deficiency Maximal incremental and endurance exercise tests in a 13 year-old patient with long-chain 3-hydroxy acyl-CoA Dehydrogenase deficiency (LCHADD) and heptanoate treatment. JIMD Rep. 2014; [Epub ahead of print]. 10.1007/8904_2014_313 PMC4241206 24997711

[13] Baruteau J, Sachs P, Broué P, Brivet M, Abdoul H, Vianey-Saban C (2012). Clinical and biological features at diagnosis in mitochondrial fatty acid beta-oxidation defects: a French pediatric study of 187 patients. J Inherit Metab Dis.

[14] Behrend AM, Harding CO, Shoemaker JD, Matern D, Sahn DJ, Elliot DL (2012). Substrate oxidation and cardiac performance during exercise in disorders of long chain fatty acid oxidation. Mol Genet Metab.

[15] Bonnet D, Martin D, Pascalede L, Villain E, Jouvet P, Rabier D (1999). Arrhythmias and Conduction Defects as Presenting Symptoms of Fatty Acid Oxidation Disorders in Children. Circulation.

[16] Gillingham MB, Connor WE, Matern D, Rinaldo P, Burlingame T, Meeuws K (2003). Optimal dietary therapy of long-chain 3-hydroxyacyl-CoA dehydrogenase deficiency. Mol Genet Metab.

[17] Gillingham MB, Weleber RG, Neuringer M, Connor WE, Mills M, van Calcar S (2005). Effect of optimal dietary therapy upon visual function in children with long-chain 3-hydroxyacyl CoA dehydrogenase and trifunctional protein deficiency. Mol Genet Metab.

[18] Gillingham M, Scott B, Elliott D, Harding C (2006). Metabolic control during exercise with and without medium-chain triglycerides (MCT) in children with long-chain 3-hydroxy acyl-CoA dehydrogenase (LCHAD) or trifunctional protein (TFP) deficiency. Mol Genet Metab.

[19] Gillingham MB, Matern D, Harding CO (2009). Effect of feeding, exercise, and genotype on plasma 3-hydroxyacylcarnitines in children with LCHAD deficiency. Topics Clin Nutr.

[20] Haglind CB, Stenlid MH, Ask S, Alm J, Nemeth A, Döbeln U (2012). Growth in LONG-CHAin 3-Hydroxyacyl-CoA Dehydrogenase deficiency. JIMD Reports.

[21] Hayes B, Lynch B, O’Keefe M, Monavari AA, Treacy EP (2007). Long chain fatty acid oxidation defects in children: importance of detection and treatment options. Ir J Med Sci.

[22] Olpin SE, Clark S, Andresen BS, Bischoff C, Olsen RKJ, Gregersen N (2005). Biochemical, clinical and molecular findings in LCHAD and general mitochondrial trifunctional protein deficiency. J Inherit Metab Dis.

[23] Sander J, Sander S, Steuerwald U, Janzen N, Peter M, Wanders RJA (2005). Neonatal screening for defects of the mitochondrial trifunctional protein. Mol Genet Metab.

[24] Saudubray JM, Martin D, de Lonlay P, Touati G, Poggi-Travert F, Bonnet D (1999). Recognition and management of fatty acid oxidation defects: a series of 107 patients. J Inherit Metab Dis.

[25] Skladal D, Sass JO, Geiger H, Geiger R, Mann C, Vreken P (2000). Complications in early diagnosis and treatment of two infants with long-chain fatty acid β-oxidation defects. J Pediatr Gastroenterol Nutr.

[26] Sperk A, Mueller M, Spiekerkoetter U (2010). Outcome in six patients with mitochondrial trifunctional protein disorders identified by newborn screening. Mol Genet Metab.

[27] Tyni T, Immonen T, Lindahl P, Majander A, Kivelä T (2012). Refined staging for chorioretinopathy in Long-Chain 3-Hydroxyacyl Coenzyme A Dehydrogenase deficiency. Ophthalmic Res.

[28] Wallimann T, Wyss M, Brdiczka D, Nicolay K, Eppenberger HM (1992). Intracellular compartmentation, structure and function of creatine kinase isoenzymes in tissues with high and fluctuating energy demands: the “phosphocreatine circuit” for cellular energy homeostasis. Biochem J.

[29] Ventura-Clapier R, Kuznetsov A, Veksler V, Boehm E, Anflous K (1998). Functional coupling of creatine kinases in muscles: species and tissue specificity. Mol Cell Biochem.

[30] Tucci S, Flögel U, Hermann S, Sturm M, Schäfers M, Spiekerkoetter U (2014). Development and pathomechanisms of cardiomyopathy in very long-chain acyl-CoA dehydrogenase deficient (VLCAD−/−) mice. Biochim Biophys Acta.

[31] Solis JO, Singh RH (2002). Management of fatty acid oxidation disorders: a survey of current treatment strategies. J Am Diet Assoc.

[32] Kinman RP, Kasumov T, Jobbins KA, Thomas KR, Adams JE, Brunengraber LN (2006). Parenteral and enteral metabolism of anaplerotic triheptanoin in normal rats. Am J Physiol Endocrinol Metab.

[33] Roe DS, Yang B-Z, Vianey-Saban C, Struys E, Sweetman L, Roe CR (2006). Differentiation of long-chain fatty acid oxidation disorders using alternative precursors and acylcarnitine profiling in fibroblasts. Mol Genet Metab.

[34] Mochel F, DeLonlay P, Touati G, Brunengraber H, Kinman RP, Rabier D (2005). Pyruvate carboxylase deficiency: clinical and biochemical response to anaplerotic diet therapy. Mol Genet Metab.

[35] Roe CR, Yang B-Z, Brunengraber H, Roe DS, Wallace M, Garritson BK (2008). Carnitine palmitoyltransferase II deficiency: successful anaplerotic diet therapy. Neurology.

